# Interaction of Protein C Inhibitor with the Type II Transmembrane Serine Protease Enteropeptidase

**DOI:** 10.1371/journal.pone.0039262

**Published:** 2012-06-19

**Authors:** Thomas A. Prohaska, Felix C. Wahlmüller, Margareta Furtmüller, Margarethe Geiger

**Affiliations:** Department of Vascular Biology and Thrombosis Research, Center for Physiology and Pharmacology, Medical University of Vienna, Vienna, Austria; Institut National de la Santé et de la Recherche Médicale, France

## Abstract

The serine protease inhibitor protein C inhibitor (PCI) is expressed in many human tissues and exhibits broad protease reactivity. PCI binds glycosaminoglycans and certain phospholipids, which modulate its inhibitory activity. Enteropeptidase (EP) is a type II transmembrane serine protease mainly found on the brush border membrane of epithelial cells in the duodenum, where it activates trypsinogen to initiate the digestion of food proteins. Some active EP is also present in duodenal fluid and has been made responsible for causing pancreatitis in case of duodeno-pancreatic reflux. Together with its substrate trypsinogen, EP is furthermore present in the epidermis and in some cancer cells. In this report, we show that PCI inhibited EP with an apparent 2nd order rate constant of 4.48×10^4^ M^−1^ s^−1^. Low molecular weight (LMWH) and unfractionated heparin (UFH) slightly reduced the inhibitory effect of PCI. The SI (stoichiometry of inhibition) value for the inhibition of EP by PCI was 10.8 in the absence and 17.9 in the presence of UFH (10 U/ml). By inhibiting trypsin, chymotrypsin, and additionally EP, PCI might play a role in the protection of the pancreas from autodigestion. Furthermore the interaction of PCI with EP may influence the regulation of epithelial differentiation.

## Introduction

Protein C inhibitor (PCI) is a serine protease inhibitor and a member of the serpin superfamily (serpinA5). PCI has originally been described as a plasma inhibitor of activated protein C (APC) [1], [2]. Later, the inhibition of several other proteases, including the pancreatic enzymes trypsin and chymotrypsin, by PCI has been shown. [3]–[12]. Like other members of the serpin family, PCI acts as a suicide substrate for its target proteases. Serpins have an exposed reactive center loop (RCL) which offers a potential cleavage site for the protease. The protease recognizes this sequence and binds to the serpin, forming a reversible Michaelis-like complex. Then the protease cleaves the reactive site peptide bond and the serpin incorporates the RCL into β-sheet A, producing a covalent serpin-protease complex [13]. The enzyme-inhibitor complex can dissociate, leaving behind an active protease and a cleaved, inactive serpin.

Heparin and other glycosaminoglycans can modify the activity and target enzyme specificity of PCI. The heparin-binding site is a basic patch on helix H, which lies close to the reactive center loop [14], [15]. Heparin changes the charge of this area, thereby modifying the affinity of PCI towards different proteases. Heparin stimulates the inhibition of APC and thrombin [16], but abolishes the inhibition of tissue kallikrein by PCI [6], [17]. Antithrombin (AT), another heparin-binding serpin, uses a different mechanism. Both low molecular weight (LMWH) and unfractionated heparin (UFH) bind to helix D. This binding leads to a conformational change of AT and an additional part of the reactive center loop is exposed. This results in increased inhibition of coagulation proteases. UFH is furthermore big enough to span from helix D to the protease. It thereby forms a template for AT and thrombin and enhances their interaction [18].

By Northern blotting, a wide tissue distribution of PCI has been demonstrated in humans. PCI mRNA is present in the liver, kidney, heart, brain, lung, spleen, reproductive system and pancreas [19], [20]. Radtke et al. showed by in situ hybridization that PCI is expressed in the exocrine part of the pancreas, and by Western blotting that the protein is present in pancreatic fluid [21]. We have shown that PCI mRNA and protein are also present in keratinocytes of the human skin [22]. Its expression is increased in the more differentiated layers of the epidermis [23]. PCI is also present in several body fluids and secretions, e.g. in plasma (∼100 nM) and seminal fluid (∼4 µM) [24]. In rodents, PCI is almost exclusively present in the reproductive tract [19]. This makes it difficult to study the effect of PCI outside the reproductive tract in animal models. Because of its wide tissue distribution, PCI may have several functions in humans. So far, very little is known about these functions. PCI might have a protective effect against cancer progression [25]–[29].

Since PCI has affinity for glycosaminoglycans and phospholipids, both components of the cell membrane, cell membrane association of PCI is not unlikely. We were therefore interested in analyzing the interaction of PCI with serine proteases also present in or on cell membranes. So far there are only a few indications in the literature, suggesting that PCI interacts with type II transmembrane serine proteases [30], [31]. However, as far as inhibition kinetics or the effect of glycosaminoglycans or phospholipids is concerned, no data is available on these interactions. It was therefore the aim of this study to analyze the interaction of PCI with enteropeptidase (EP; other names: enterokinase, PRSS7, TMPRSS15). EP (EC 3.4.21.9) is a type II transmembrane serine protease, located mainly at the brush border membrane of the epithelial cells of the duodenum and jejunum [32]. Active EP also occurs in duodenal fluid [33]. In the small intestine, EP activates trypsinogen to trypsin [33]. Active human EP is composed of a light and a heavy chain linked by a disulfide bond [33]. The catalytic center is located on the light chain, whereas the heavy chain is responsible for substrate specificity [34].

Activation of trypsinogen is an obligatory step in the pathogenesis of acute necrotizing pancreatitis [35]. So far, it is not fully understood how trypsinogen is activated prematurely *in vivo*. This function might be executed intracellularly by cathepsin B [36]. Some authors also suggest a role of EP by reflux of duodenal fluid into the pancreatic duct [37]. However, this theory remains controversial. Outside the digestive system, EP and its substrate trypsinogen are present in keratinocytes during their terminal differentiation and might be involved in the regulation of desquamation [38]. They are also expressed by oral squamous cell carcinoma (HSC-3) and prostate cancer (PC-3) cell lines [39], [40]. Active trypsin can increase tumor cell invasiveness [41], [42]. Regulation of EP by protease inhibitors may therefore be not only important in the digestive system, but also in epidermal differentiation and tumor invasion.

We analyzed the interaction of EP with serpin-type protease inhibitors (PCI, α_1_-antitrypsin (A1AT) and AT) and can show that PCI is a strong inhibitor of EP with an apparent 2^nd^ order constant (k_app_) comparable to other PCI-protease interactions.

## Results

### Interaction of Human EP with PCI Leads to Complex Formation

**Figure 1:**
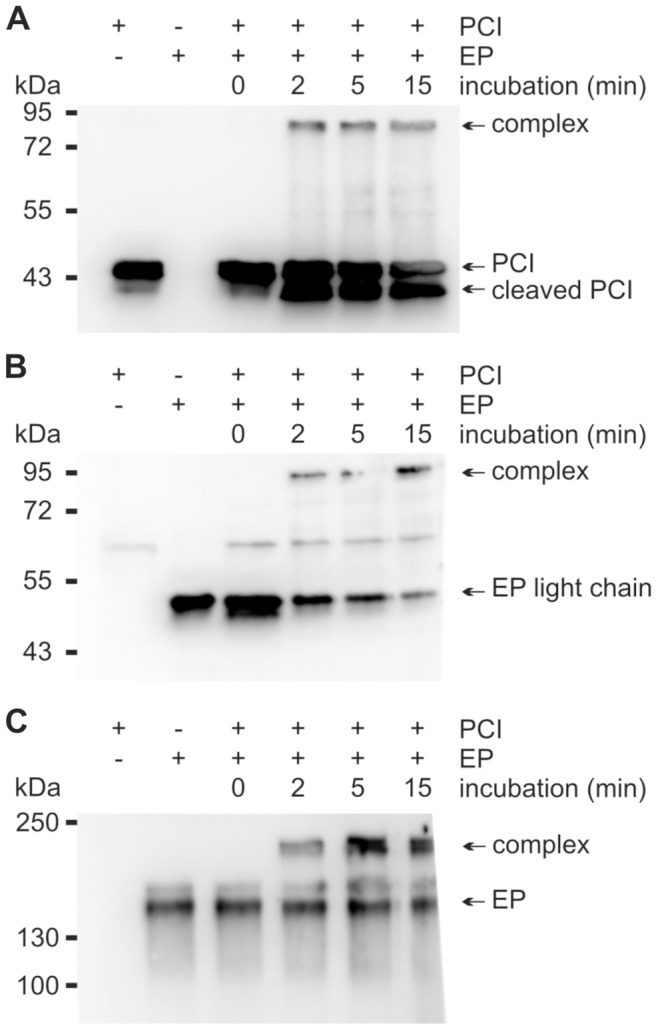
PCI forms complexes with EP and is cleaved by EP. Recombinant human PCI (440 nM) and EP (60 nM) were incubated at 37°C in a volume of 10 µl TCNB buffer for the time periods indicated. Thereafter, an equal amount of Laemmli buffer with (**A, B**) or without (**C**) 10% 2-mercaptoethanol was added. For time point zero, the reagents were applied directly into Laemmli buffer. SDS-PAGE and Western blotting were performed as described in Materials and Methods using either monoclonal mouse anti-PCI IgG (**A**), rabbit anti-EP (light chain) IgG (**B**) or mouse anti-EP (heavy chain) IgG (**C**), respectively. The position of the molecular mass markers (kDa) is indicated on the left. For control purposes, the left 2 lanes contained PCI alone or EP alone, respectively.

The interaction between recombinant human PCI and EP was analyzed by SDS-PAGE followed by Western blotting using antibodies to PCI and EP, respectively. Upon incubation of PCI with EP, an increase in the intensity of the band corresponding to the cleaved form of PCI and complex formation was seen (Figure 1A). Under reducing conditions, the complex had a molecular mass of about 95 kDa. This suggests an interaction of PCI (46 kDa) with the light chain of EP (∼50 kDa), on which the serine protease domain is localized. To confirm this finding, we used an antibody to the light chain of EP. We could observe complex formation comparable to the results when anti-PCI IgG was used (Figure 1B). We performed the same experiment under non-reducing conditions, using an antibody to the heavy chain of EP. Here, it was possible to see complex formation at about 215 kDa (Figure 1C).

### Effect of Cofactors on the Inhibition of EP by PCI

**Figure 2:**
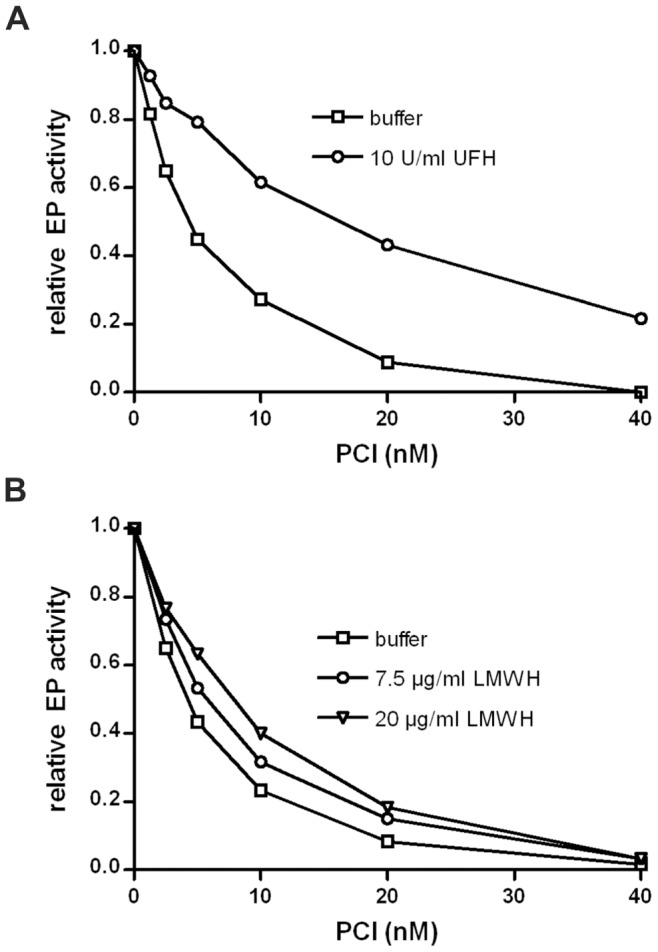
Inhibition of EP by PCI in the absence or presence of heparin. EP (0.3 nM) was incubated with different concentrations of recombinant human PCI (as indicated) for 45 min at 37°C in the absence or presence of either UFH (**A**) or LMWH (**B**) at the indicated concentrations. Remaining EP activity was quantified using Z-Lys-SBzl as described in Materials and Methods. The activity of EP in the absence of PCI was set as 1. Each value represents the mean of duplicates. Similar results where obtained with different preparations of human and mouse PCI.

In the EP activity assay, PCI strongly inhibited the amidolytic activity of EP (Figure 2). UFH (Figure 2A) and LMWH (Figure 2B) slightly reduced the inhibitory effect of PCI. Inhibition of EP with purified plasma PCI showed similar results as with recombinant PCI (data not shown). The phospholipid PAPS (1-palmitoyl-2-arachidonoyl-sn-glycero-3-phospho-L-serine) had no effect on the inhibition of EP by PCI. Consistent with previous results [43], PAPS strongly increased the inhibition of activated protein C in a parallel experiment. Recombinant mouse PCI showed a similar inhibitory effect on human EP as human PCI. With mouse PCI, the inhibitory effect was also slightly reduced in the presence of LMWH (data not shown).

**Figure 3:**
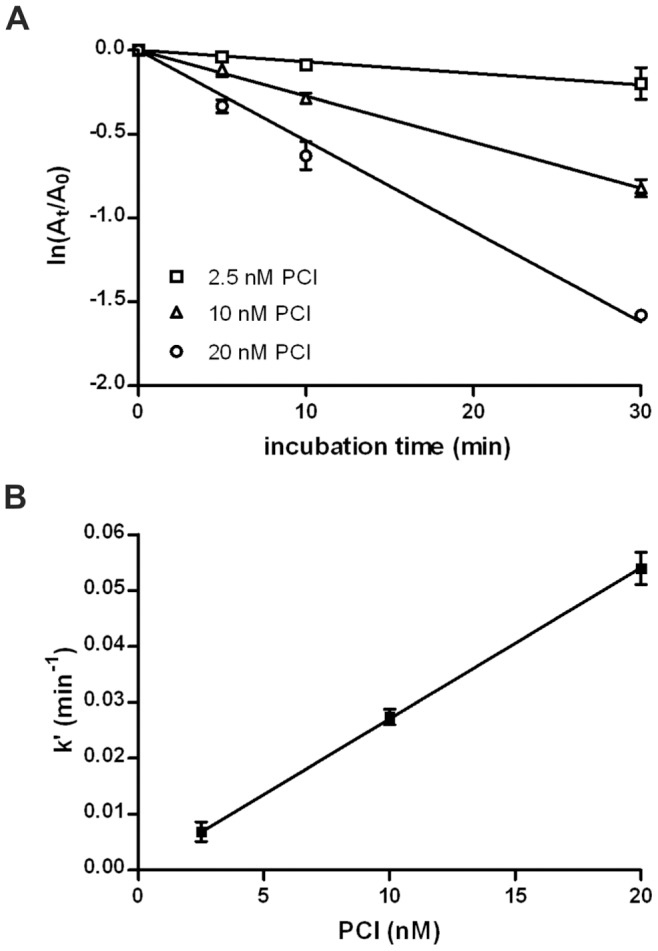
Determination of the apparent 2^nd^ order rate constant for the inhibition of EP by PCI. EP (0.3 nM) was incubated with indicated concentrations of recombinant human PCI for different periods of time. Remaining enzyme activity was determined as described in Materials and Methods and plotted as ln(A_t_/A_0_) versus incubation time (**A**). For each concentration, the negative slope of the linear regression was calculated. This value represents the respective pseudo 1^st^ order constant (k’). k’ was then replotted against PCI concentration (**B**). Linear regression was used to obtain the slope of the regression line which represents the apparent 2^nd^ order rate constant (k_app_). The k_app_ was calculated for each experiment (n = 3) separately. Each single experiment was performed in duplicates. This yielded a k_app_ of 4.48±0.32×10^4^ M^−1^ s^−1^ (mean ± S.D., n = 3). In the presence of 10 U/ml UFH, the k_app_ was 8.83±0.75×10^3^ M^−1^ s^−1^ (mean ± S.D., n = 3). All 3 experiments for calculating the k_app_ in the absence of UFH were integrated into the figure. **A:** Each value represents the mean of 3 independent experiments, each in duplicates. Error bars show standard deviation. **B:** Error bars show the 95% confidence interval of the respective k’.

**Figure 4:**
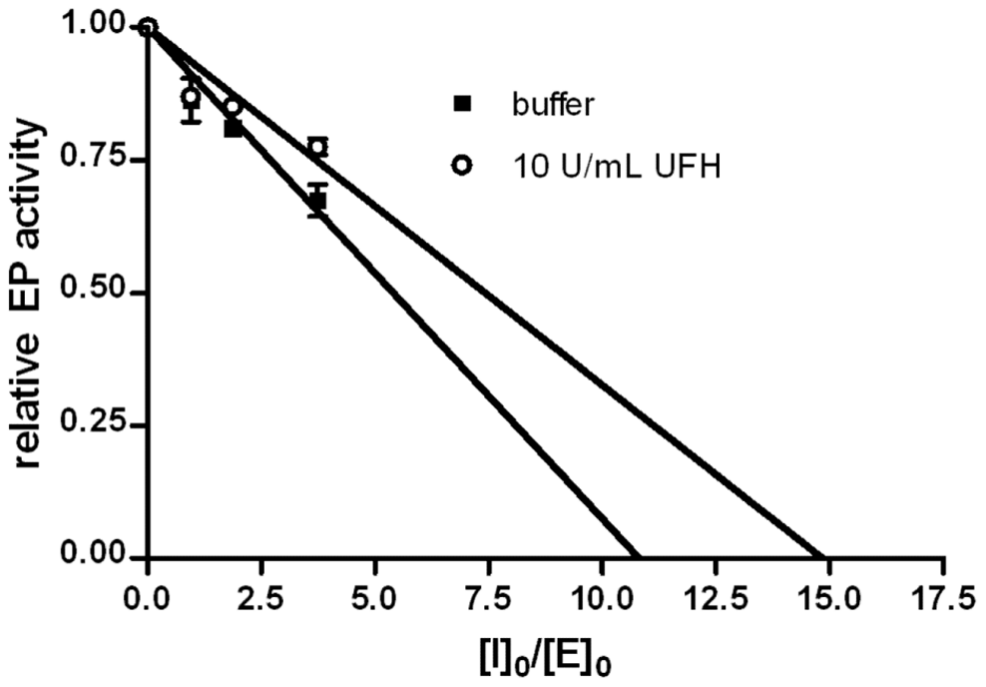
Stoichiometry of inhibition of EP by PCI. EP (0.3 nM) was incubated overnight with varying concentrations of recombinant human PCI in the absence and presence of 10 U/ml UFH. Remaining EP activity was determined and plotted as relative activity against [PCI]_0_/[EP]_0_. The x-intercept of each linear regression line represents the SI value. The SI values were 10.8±0.7 without and 17.9±0.6 (mean ± S.D., n = 3) with 10 U/ml UFH, respectively. The results of one experiment are shown in the figure. Each single experiment was done in duplicates.

The apparent 2^nd^ order rate constants (k_app_) for the inhibition of EP by human recombinant PCI were calculated under pseudo 1^st^ order conditions as outlined in Materials and Methods (Figure 3). PCI inhibited EP with an apparent 2^nd^ order rate constant of 4.48±0.32×10^4^ M^−1^ s^−1^ (mean ± S.D., n = 3). In the presence of 10 U/ml UFH, the constant was reduced to 8.83±0.75×10^3^ M^−1^ s^−1^ (mean ± S.D., n = 3). The SI value for the inhibition of EP by PCI was 10.8±0.7 (mean ± S.D., n = 3). In the presence of 10 U/ml UFH, it increased to 17.9±0.6 (mean ± S.D., n = 3) (Figure 4). The k_app_ values were corrected for SIs by multiplication (k_app_ × SI), as this provides a measure for the overall rate of association [13], [44]. Corrected rate constants (k_app_ × SI) were 4.84×10^5^ M^−1^ s^−1^ in the absence and 1.58×10^5^ M^−1^ s^−1^ in the presence of 10 U/ml UFH, respectively.

### Human PCI Forms Complexes with EP from Natural Sources

**Figure 5:**
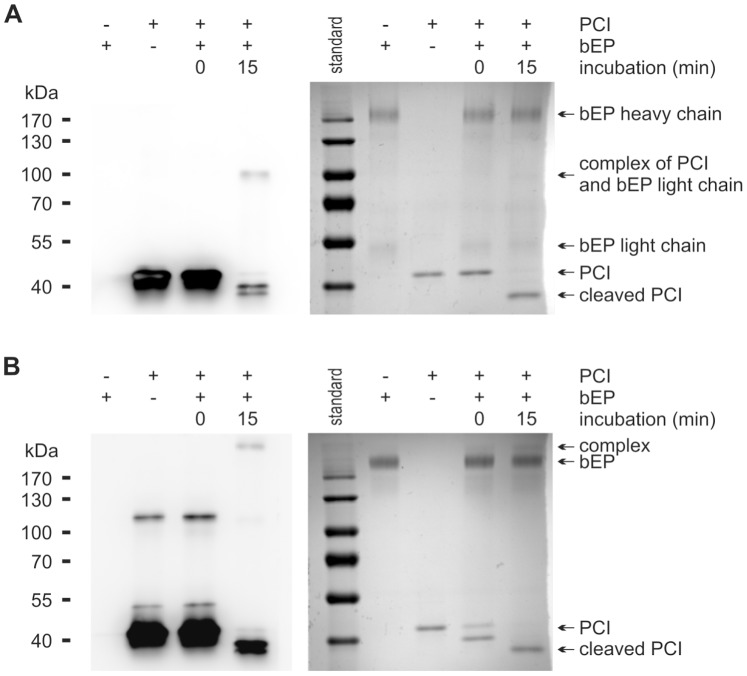
Bovine EP cleaves human PCI and complex formation is observed. PCI (0.05 g/l) and bovine EP (0.04 g/l) were incubated at 37°C in a volume of 10 µl TCNB buffer for the time periods indicated. Thereafter, an equal amount of Laemmli buffer with (**A**) or without (**B**) 10% 2-mercaptoethanol was added. For time point zero, the reagents were applied directly into Laemmli buffer. SDS-PAGE and Western blotting using mouse anti-PCI IgG (**left panel in A and B**) or SDS-PAGE followed by Coomassie staining (**right panel in A and B**) using PageBlue solution (Fermentas, St. Leon-Rot, Germany) were performed as described in Materials and Methods. The position of the molecular mass standard (kDa) is indicated on the left and also visible as first lane on the right panel in A and B.

**Figure 6:**
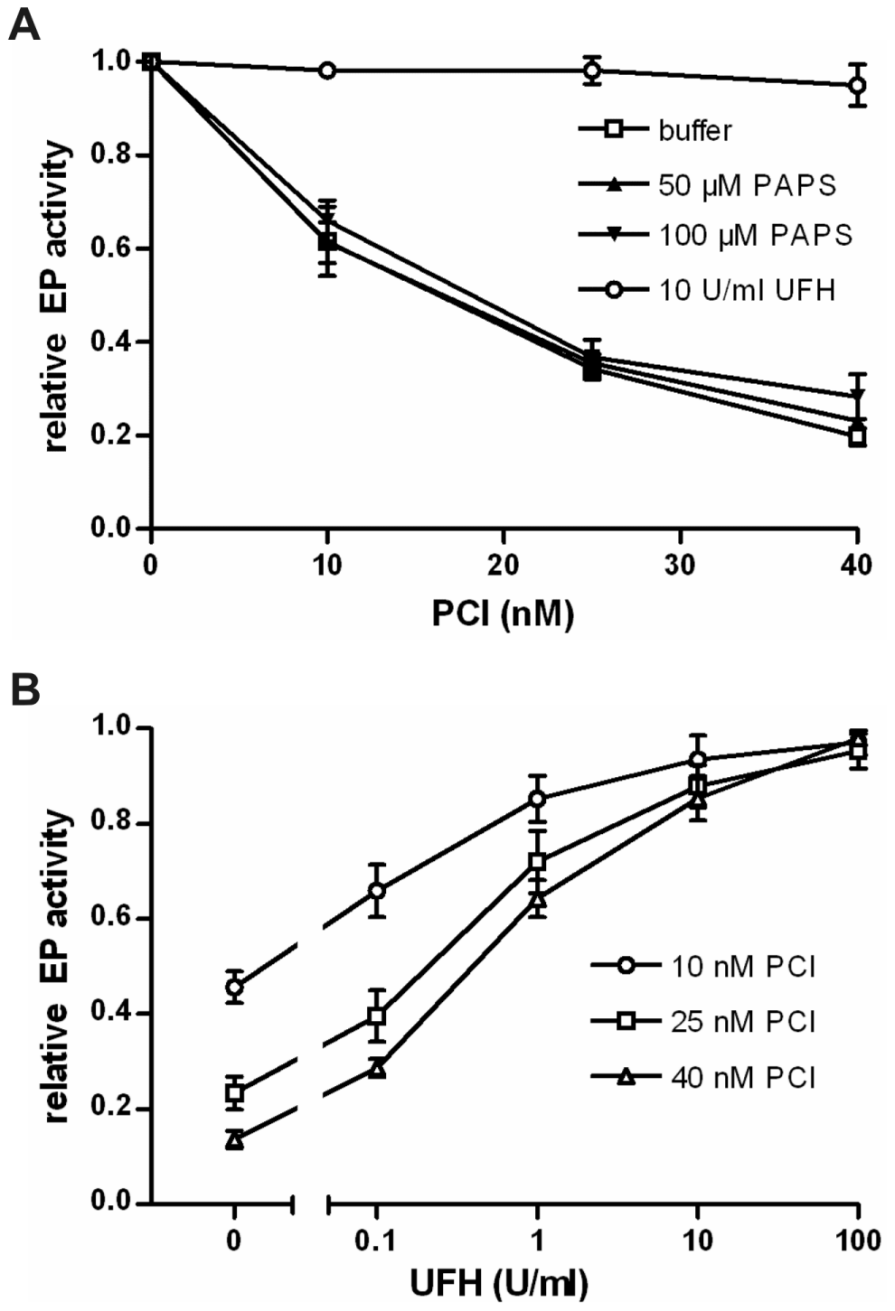
Inhibition of bovine EP by PCI in the absence or presence of UFH and phospholipid vesicles. Bovine EP (0.05 µg/ml) was incubated with different concentrations of PCI (as indicated) for 30 min at 37°C in the absence or presence of either UFH or PAPS at the indicated concentrations (**A**). In an additional activity assay for bovine EP, PCI (10–40 nM) and UFH (0.1–100 U/ml) were added and incubated at 37°C for 45 min. Increasing concentrations of UFH reduced the inhibitory activity of PCI towards EP (**B**). Remaining EP activity was quantified using Z-Lys-SBzl as described in Materials and Methods. The activity of EP in the absence of PCI was set as 1. n = 3 (for each figure); error bars show standard deviation. Each single experiment was done in duplicates.

In addition to recombinant human EP, the interaction of PCI with two additional EPs from natural sources has been studied. EP purified from porcine intestine (data not shown) as well as bovine EP (bEP) purified from calf intestine formed complexes with PCI, as demonstrated by SDS-PAGE analysis followed by Western blotting (Figure 5). Functional assays showed strong inhibition of bEP by PCI. The presence of PAPS did not affect the inhibitory property of PCI towards bEP (Figure 6A), which is consistent with results obtained with human EP.

A dose-response curve for the effect of UFH on the inhibition of bEP by PCI was determined. Increasing concentrations of UFH strongly reduced the inhibitory activity of PCI towards bEP (Figure 6B). The interfering effect of UFH on bEP inhibition by PCI was even stronger as compared with the inhibition of human recombinant EP.

### Interaction of Other Serpins with Human EP

**Figure 7:**
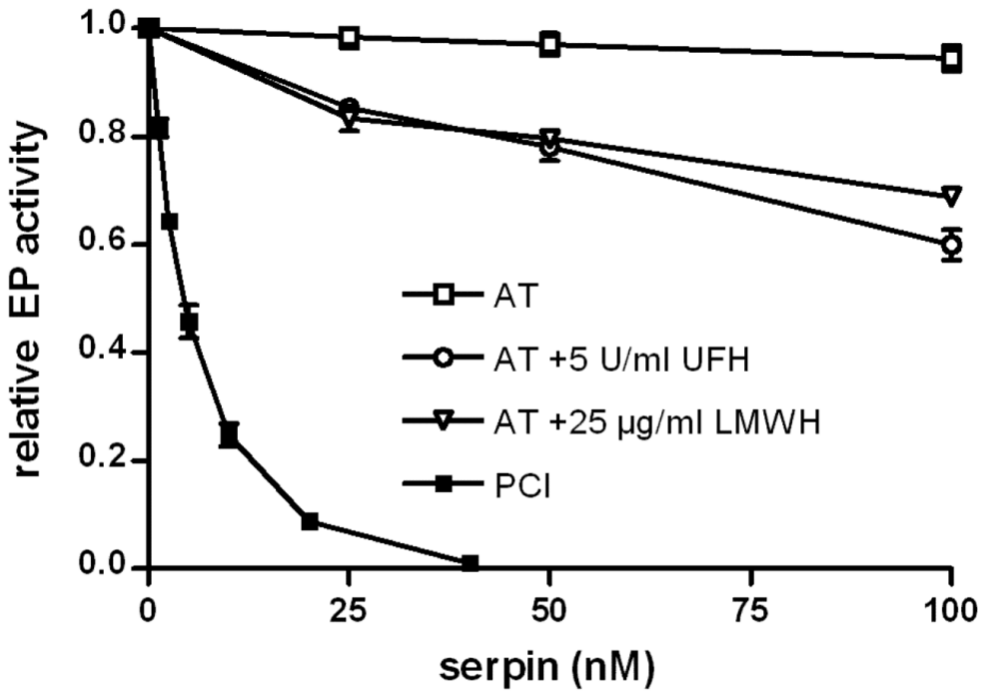
Inhibition of EP by antithrombin. EP (0.3 nM) was incubated for 60 min at 37°C with varying concentrations of AT in the absence and presence of UFH or LMWH (as indicated in the figure). For comparison, inhibition by PCI (incubation time 45 min) is also included. Remaining EP activity was quantified as described in Materials and Methods. The activity of EP in the absence of serpins was set as 1. n = 3; error bars show standard deviation. Each single experiment was done in duplicates.

To investigate if other serpins also interact with EP, activity assays were performed in the presence of either A1AT or AT. After incubation of EP with A1AT for 60 min, no significant reduction of EP activity was observed, even at a molar [A1AT]:[EP] ratio of 1000∶1 (not shown). When EP was incubated with AT for the same time, there was no reduction in EP activity. However, AT slightly inhibited EP when either UFH or LMWH were present (Figure 7). Heparin alone had no effect on the activity of EP. Inhibitory activity of A1AT and AT was assured by experiments studying inhibition of trypsin by A1AT and of thrombin by AT, respectively.

### Western Blotting of Pancreas Lysate

Human pancreas lysate was applied to SDS-PAGE and Western blotting. Monoclonal anti-serpinA5 IgG was used to detect PCI. A band at about 57 kDa was seen in pancreas lysate, corresponding to the molecular mass of PCI purified from human plasma. Additionally, two faint bands at about 30 and 37 kDa (possible degradation products) were seen (not shown).

## Discussion

In this study, we can demonstrate that PCI is a fairly strong inhibitor of EP, with a k_app_ comparable to most protease-PCI interactions which range from 8.00×10^2^ M^−1^ s^−1^ for APC inhibition in the absence of heparin to 5.60×10^7^ M^−1^ s^−1^ for acrosin inhibition in the presence of heparin [9], [45]. It was the first time that an interaction of EP with a serpin-type inhibitor was shown. Additionally, it was also the first time that inhibition rate constants and the stoichiometry of inhibition were calculated for the interaction of a transmembrane serine protease with PCI.

It has been shown previously that heparin and phospholipids are able to stimulate or to reduce the inhibitory activity of PCI towards several proteases [43], [46]. Glycosaminoglycans like heparin seem to regulate the inhibitory activity of PCI by binding to the target protease as well as to the serpin [13]. In case of PCI, this bridging mechanism is strongly protease-dependent and often leads to enhancement of protease inhibition [47]. Interestingly, the inhibition of plasma kallikrein by PCI is not stimulated by heparin [3], factor Xa inhibition shows only a slight stimulation [48], and the interaction of PCI with tissue kallikrein is completely abolished in the presence of glycosaminoglycans [6]. Heparin slightly reduced the inhibition of recombinant human EP by PCI and this effect was even more pronounced using bovine EP purified from calf intestine. This could be explained by the fact that the recombinant EP carries a positively charged His-tag at the C-terminus which might counteract the repulsive effect of the negatively charged heparin.

AT, on the other hand, inhibited EP only when heparin was present. So, heparin stimulated the inhibition of EP by AT, but reduced the inhibition of EP by PCI. To our knowledge, this is the first demonstration that heparin led to a reduced inhibition of a particular protease by PCI, but an increased inhibition by AT. This may be due to differences in regulation of serpin activity by heparin, as AT undergoes a conformational change when bound to glycosaminoglycans compared to the bridging mechanism of PCI [47]. Furthermore the different heparin-binding sites of PCI (helix H) and AT (helix D) may also contribute to this opposed effect [14], [18].

As mentioned above, native EP is a type II transmembrane serine protease. It contains an N-terminal hydrophobic segment from position 18 to 44, predicted to span the membrane [49], [50]. The recombinant EP used is a mixture of two forms, in which the heavy chain is truncated and starts either at Leu41 or Ser118. N-terminal sequence analysis by Edman degradation revealed that also the bEK contains a mixture of two heavy chains starting at Gly53 and Ser118 respectively (personal communication: Dr. B. Sarg, Innsbruck Medical University, Austria). Phospholipids did not influence EP inhibition by PCI. Assuming a heparin-like bridging mechanism for the stimulatory effect of phospholipids on PCI-protease interactions, these results are not surprising, since it has been shown previously that a truncated EP lacking the transmembrane domain does not interact with phospholipid vesicles [51]. Supporting this data, a commercially available protein-lipid overlay assay containing membrane phospholipids was performed. We could not detect any binding of recombinant human EP to phospholipids (unpublished data). From our data it cannot be excluded that the interaction of PCI with the catalytically active light chain of EP is influenced by membrane anchoring of EP. However, the huge heavy chain lies in between the active center and the plasma membrane. This could hinder potential phospholipid-bridging of PCI and the light chain of EP. It is therefore not very likely that phospholipids involved in anchoring of EP could represent a bridge for bringing together PCI and EP.

Several publications have shown that PCI mRNA is highly expressed in the pancreas [19], [20], particularly in the exocrine part [21]. We could show by Western blotting that PCI protein is present in human pancreas lysate. However, we were not able to show its presence in the exocrine part by immunohistochemistry on paraffin-embedded tissue sections.

Activation of trypsinogen is a crucial step in the pathogenesis of necrotizing pancreatitis [35]. So far, it is not fully understood how trypsinogen is activated prematurely within the pancreas. Some authors believe that this activation might origin from reflux of EP-containing duodenal fluid into the pancreatic duct [37]. Though, this theory remains controversial and it is not clear if duodenopancreatic reflux occurs *in vivo* and, if it does, whether it is really able to damage the pancreas profoundly. However, if duodenopancreatic reflux occurs under some circumstances, inhibition of EP by PCI might have a protective effect. In addition, PCI could protect the pancreas from autodigestion by inhibiting trypsin and chymotrypsin [10]. Polymorphisms of PSTI (pancreatic secretory trypsin inhibitor) are associated with a higher incidence of pancreatitis [52], suggesting that this inhibitor may have a protective effect against this disease. Polymorphisms of PCI have been investigated with respect to male fertility [53]. Further studies are needed to address the question if polymorphisms of PCI might also be associated with pancreatitis.

PCI has also been identified in the epidermis, with increasing amounts in its more superficial layers [22], [23]. Desquamation of keratinocytes is controlled by several proteases and their inhibitors. An imbalance between them can lead to inflammatory skin diseases such as psoriasis [54]. Since both, EP and PCI, are expressed in the upper epidermis [22], [38], the interaction of EP and PCI might also be involved in the regulation of epidermal differentiation.

The SI value (10.8) of the PCI/EP-interaction is rather high, especially in the presence of heparin (17.9), suggesting that a significant amount of PCI is cleaved/inactivated by EP. The biological consequences of the PCI/EP-interaction may depend on the local [PCI]:[EP] ratio. At a high [PCI]:[EP] ratio, the inhibition of EP would be nearly complete leading to impaired activation of trypsinogen. Additionally, some active PCI would still be available to inhibit trypsin directly, further reducing its activity. At a low [PCI]:[EP] ratio, the available PCI would not be sufficient to completely inhibit EP. Remaining active EP would still be able to activate trypsinogen and to cleave PCI.

For the digestive system, this could mean that in the pancreatic duct, any possibly present EP could be efficiently inhibited by PCI. In the duodenum, where EP is abundantly present, PCI would have only a minor effect on EP activity and would not significantly interfere with the activation of digestive proteases.

In the epidermis, where EP is mainly localized in the superficial layers [38], any EP present in more basal layers would be efficiently inhibited by excess PCI. In more superficial layers, EP would be able to activate trypsinogen in significant amounts, so it can contribute to the desquamation process.

## Materials and Methods

### Materials

Expression in *Escherichia coli* and purification of recombinant human and mouse PCI was performed as described previously [55]. Citrated normal human plasma was obtained from the Austrian Red Cross and PCI was purified as described [43]. Recombinant human EP and monoclonal mouse anti-serpinA5 IgG were obtained from R&D Systems (Minneapolis, MN). EP purified from calf intestine was from Roche (Basel, Switzerland). Purified human plasma AT and A1AT, thermolysin, 1,10-phenantroline, Ellman’s reagent, purified porcine EP and dry milk were from Sigma-Aldrich (St. Louis, MO). Z-Lys-SBzl was from Bachem (Bubendorf, Switzerland). LMWH was from Santa Cruz Biotechnology (Santa Cruz, CA). PAPS was from Avanti Polar Lipids (Alabaster, AL). UFH was from Baxter (Vienna, Austria). PVDF membranes were from Millipore (Billerica, MA). SuperSignal West Femto was from Thermo Scientific (Rockford, IL). Monoclonal anti-PRSS7 IgG was from Abgent (San Diego, CA). Rabbit anti-EP-light-chain IgG was a kind gift from the lab of J.E. Sadler (Washington University School of Medicine, St. Louis, MO). HRP-conjugated donkey anti-rabbit and sheep anti-mouse IgG were from GE Healthcare (Waukesha, WI). Human pancreas lysate was from Zyagen (San Diego, CA).

### Preparation of Phospholipid Vesicles

Phospholipid vesicles were prepared freshly for each experiment as described previously [43]. In brief PAPS was dissolved in chloroform (10 g/l) and stored at −80°C. Different aliquots of PAPS solution were pipetted into Eppendorf tubes and chloroform was evaporated using argon. Prewarmed buffer (37°C) was used to resuspend dried PAPS by vortexing at maximal speed for 60 seconds followed by shaking (1400 rpm) for 5 minutes at 37°C.

### Activity Assay for EP

Recombinant human EP (100 µg/ml) was activated by incubation with thermolysin (3.16 µg/ml) in TCNB buffer (50 mM Tris, 0.15 M NaCl, 10 mM CaCl_2_, and 0.05% Brij-35, pH 7.5). After 30 min, the reaction was stopped by adding 10 mM 1,10-phenantroline.

To determine EP activity, Z-Lys-SBzl was used at 200 µM in TCNB containing 200 µM Ellman’s reagent (5,5′-dithiobis-(2-nitrobenzoic acid)). The absorption was recorded at 405 nm for 5 min to assess the activity of EP. The final concentration of human EP was 0.3 nM in all assays. To study the inhibition of EP by PCI and other serpins, EP was incubated with different concentrations of the respective serpin for different periods of time (5 to 60 min) at 37°C in a total volume of 100 µl TCNB buffer. In some reactions, UFH (0.1–100 U/ml), LMWH (0.5–20 µg/ml), or PAPS (100 µM) were included. The reactions were stopped by the addition of an equal amount of substrate solution. The specific activity of EP was 32.1±2.8 nmol/µg/min (mean ± S.D., n = 6).

Apparent 2^nd^ order rate constants for the inhibition of EP were determined as described previously for the inhibition of other target enzymes by PCI [6], [8]. The stoichiometry of inhibition (SI) was determined by incubating PCI (0.28–2.21 nM) and EP (0.3 nM) overnight at 37°C in TCNB buffer. Afterwards, an activity assay was performed. The relative EP activity was plotted against the [PCI]_0_/[EP]_0_ ratio. Linear regression was used to calculate the SI value (x-intercept of the linear regression line).

Lyophilized bovine EP (bEP) was reconstituted to a concentration of 0.5 mg/ml and stored in aliquots at −80°C until further usage. The final concentration of bEP was 0.05 µg/ml and the activity was determined as described for human EP. 1,10-phenanthroline (used to stop the activation of human recombinant EP) had no effect on the inhibition of bovine EP by PCI (data not shown).

### SDS-PAGE and Western Blotting

SDS-PAGE was performed according to the method of Laemmli [56] using 10% acrylamide gels. EP (60 nM) and PCI (440 nM) were preincubated in 10 µl TCNB buffer for varying times at 37°C. The reactions were stopped by the addition of an equal volume of Laemmli buffer (125 mM Tris, 20% glycerol, 4.1% SDS, 0.01% bromphenol blue with or without 10% 2-mercaptoethanol) and heating the samples for 5 min at 95°C. For the zero-time point the proteins were added directly into Laemmli buffer. Thereafter, they were applied to a 10% acrylamide gel. After electrophoresis, the gels were transferred to PVDF membranes for Western blotting. Blocking of membranes was done with 5% dry milk in PBST (135 nM NaCl, 1.3 mM KCl, 2.5 mM Na_2_HPO_4_, 0.5 mM KH_2_PO_4_, 0.1% Tween-20) either at 4°C overnight or at room temperature for 1 h. Incubations with the primary antibody were also done in 5% milk in PBST at 4°C overnight or at room temperature for 1 h. The membranes were washed 5 times (5 min each) in PBST. The secondary peroxidase-labeled antibody was applied for 1 h in PBST with 5% dry milk. After another washing step, the peroxidase reaction was detected with SuperSignal West Femto. Primary antibodies used in Western blotting were monoclonal mouse anti-serpinA5 IgG (0.5 µg/ml), monoclonal anti-PRSS7 (enteropeptidase) IgG (1 µg/ml) and polyclonal rabbit anti-EP-light-chain IgG (78 µg/ml). The respective secondary antibodies were HRP-conjugated donkey anti-rabbit IgG (0.11 µg/ml), and HRP-conjugated sheep anti-mouse IgG (0.13 µg/ml).
